# One-Year Visual and Refractive Outcomes following LASIK for Myopia and Myopic Astigmatism with MEL 90 versus Schwind Amaris 750S Excimer Laser: A Comparative Study

**DOI:** 10.1155/2021/9929181

**Published:** 2021-06-25

**Authors:** Sheetal Brar, Dishitha P. Rathod, C. R. Roopashree, Sri Ganesh

**Affiliations:** Nethradhama Super Speciality Eye Hospital, Bangalore, India

## Abstract

**Purpose:**

To compare clinical outcomes following LASIK for myopia performed with MEL 90 vs. Schwind Amaris 750S excimer laser.

**Methods:**

Data were collected retrospectively for patients who underwent Femto-LASIK, using the MEL 90 and Schwind Amaris 750S excimer laser for correction of myopia and myopic astigmatism within the range of −1.00 to −10.00 D SE from January 2013 till June 2018. Outcomes were analysed at 12 months for safety, efficacy, enhancement rate, and long-term complications.

**Results:**

A total of 328 eyes of 328 patients were analysed. One hundred and sixty-three eyes were treated with Schwind Amaris and the remaining 165 eyes with the MEL 90 laser. Twelve months postoperatively, the mean UDVA, CDVA, residual SE, and cylinder in the Amaris group were −0.10 ± 0.09 logMAR, −0.14 ± 0.06 logMAR, −0.21 ± 0.22 D, −0.13 ± 0.18 D versus −0.05 ± 0.07 logMAR, −0.09 ± 0.08 logMAR, −0.23 ± 0.23 D, and −0.14 ± 0.21 D for the MEL 90 group (*p* values >0.05). For the Amaris group, safety and efficacy indices were 1.12 and 1.02, whereas for the MEL 90 group, these indices were 1.08 and 1.00, respectively. No eye in either group had any postop flap-related complications, infectious keratitis, diffuse lamellar keratitis, or keratectasia. Two eyes in the Amaris and 4 eyes in MEL 90 group required enhancement for the progression of myopia.

**Conclusion:**

At 12 months, both Schwind Amaris 750S and MEL 90 lasers demonstrated comparable clinical outcomes for myopic LASIK in a single surgeon setting.

## 1. Introduction

Laser in situ keratomileusis (LASIK) is one of the most widely performed laser vision correction surgery worldwide, clinical results of which have improved over the past decade due to significant advancements in techniques and technology. The introduction of femtosecond laser flap creation vastly reduced microkeratome-related complications and improved the safety and efficacy of LASIK [1, 2]. Newer generations of excimer laser machines have also contributed to improved results of LASIK in recent years, due to the use of scanning beams or flying spots, with smaller spot sizes and more efficient eye trackers [3, 4].

The MEL 90 excimer laser (Carl Zeiss Meditec, Jena, Germany) is an upgrade to its predecessor, the MEL 80, with advanced features such as faster pulse rate, compatibility with the new Triple-A ablation profile, and further improved dynamic flow cone for controlled atmosphere [5]. The safety and efficacy of MEL 90 laser have already been evaluated for the treatment of myopia, hyperopia, and mixed astigmatism [5–7]; however, no comparison study has been reported so far, comparing its outcomes with any of the existing excimer lasers.

The aim of this study was to evaluate and compare the visual and refractive results of myopic Femto-LASIK performed with the MEL 90 versus Schwind Amaris 750 excimer laser platforms; both the platforms are currently being claimed as the fastest excimer lasers [7–9]. We also wanted to test the hypothesis that a faster ablation rate might lead to better predictability in the outcomes.

## 2. Materials and Methods

This was a retrospective, comparative study of all patients who underwent Femto-LASIK for myopia or myopic astigmatism at Nethradhama Superspeciality Eye Hospital, Bangalore, between January 2015 and June 2018, using either MEL 90 or Schwind Amaris 750s excimer laser system. Ethics committee approval was not deemed necessary due to the retrospective nature of the study. Data were retrieved from the electronic medical records and both groups were matched for age and preoperative refractive error.

All patients had undergone a complete preoperative ophthalmic evaluation including manifest and cycloplegic refraction, corneal topography with Pentacam Scheimpflug imaging (OCULUS, Optikgerate GmbH, Wetzlar, Germany) & Orbscan topographer (Orbscan IIz, Bausch & Lomb), slit lamp, dry eye evaluation, and indirect ophthalmoscopy for dilated fundus examination.

Inclusion criteria were as follows: (1) myopia or myopic astigmatism in the range of −1.00 to −10.00 D spherical equivalent (SE); (2) manifest cylinder up to −6.00 D; (3) stable refractive error for the past 12 months (change in SE of <0.5 D); (4) corrected distance visual acuity (CDVA) of 20/30 or better. Exclusion criteria were the usual ones followed for case selection for corneal LASIK surgery [8].

Following thorough counselling, informed consent was obtained from each patient. Patients had surgery with either of the two excimer lasers available at our center, MEL 90 or Schwind Amaris 750S. All surgeries were performed by a single, experienced, high volume refractive surgeon (SG) using a standard technique of Femto-LASIK.

### 2.1. Schwind Amaris 750S

The Schwind Amaris 750S laser (SCHWIND eye-tech-solutions GmbH & Co. KG, Kleinostheim, Germany) is a flying spot laser working at a true repetition rate of 750 Hz and produces a beam size of 0.54 mm FWHM (full width at half maximum) with a super-Gaussian ablative spot profile. High-speed eye-tracking (pupil and limbus tracker with cyclotorsional tracking) with a 1050 Hz acquisition rate is accomplished with a 3 ms latency time.

The Amaris 750S uses a dual-fluence concept. Approximately the first 80% of the ablation is performed with higher pulse energy, and the last 20% is completed with lower pulse energy to achieve a smooth ablation surface. Its Intelligent Thermal Effect Control prevents damage to the surrounding corneal tissue because the laser pulses are distributed in a thermally optimized, dynamically adapted way, giving each position on the cornea sufficient time to cool down before being hit by another laser pulse [8–10].

### 2.2. MEL 90

The MEL 90 (Carl Zeiss Meditec, Jena, Germany) uses a Triple-A ablation profile, which integrates the original MEL 80 Aberration Smart Ablation (ASA) profile for low myopic corrections and Tissue Saving Ablation (TSA) profile for high myopic corrections into a single profile, to reduce the ablation depth. The platform provides the option to operate at 250 Hz (the same frequency as the MEL 80) or 500 Hz, a feature known as “Flexiquence.” The infrared eye tracker operates at 1,050 Hz, tracks the pupil border and the corneal limbus, and can be offset manually so that the treatment may be centered on the coaxially sighted corneal light reflex rather than to the entrance pupil center. The small 0.7 mm Gaussian flying spot and the nonrandom proprietary shot distribution pattern ensure that corneal heating is kept below the relevant threshold so that the 500 Hz pulse rate can be safely used continuously for the whole ablation to avoid overheating of the corneal surface [5, 6].

### 2.3. Treatment Planning

In the Schwind Amaris group, ablation calculation and treatment planning were done using an aspheric aberration neutral (Aberration-Free™) with the ORK-CAM software module, which enables automatic iris registration for cylinders.

In the MEL 90 group, for eyes with cylinder ≤1.00 D, the treatment was directly planned on the MEL 90 laser at 500 Hz pulse frequency using a Triple-A profile, which is an aspherically optimized ablation profile and allows for a wide range of spherocylindrical (SCA) corrections including eyes with higher and lower levels of ametropia, simplifying the treatment planning. However, in eyes with >1.00 D, Wavefront Supported Customized Ablation (WASCA) aberrometry was performed for iris registration and treatment plan using the CRS-Master software and imported into the laser. The treatment profile used for these eyes was Aberration Smart Ablation (ASA), and the laser pulse frequency used was 250 Hz, as the laser allows only a frequency of 250 Hz to be used for customized treatments.

### 2.4. Surgical Protocol

All treatments in both the groups were performed as bilateral simultaneous Femto-LASIK using the VisuMax femtosecond laser for flap creation at 110 microns. Scotopic pupil diameter, along with the amount of myopia being treated, was used to choose the optical zone within the pachymetric safety limits. No nomogram adjustments were used in either group. All cases underwent a fluency test daily prior to the procedure and were uneventful. No eye in either group had any intraoperative complications such as suction loss, dense opaque bubble layer, gas breakthrough, flap tears, etc., requiring postponing or abandoning of the procedure.

Postoperative medications were the same for all patients and included a combination of 0.5% moxifloxacin ophthalmic solution (Vigamox®; Alcon) and 0.1% prednisolone (Predforte®; Allergan) eyedrops in a tapering dose for 10 days and installation of preservative-free artificial tear supplements 4 times a day for a month.

### 2.5. Postoperative Evaluation

Patients were examined on postoperative day 1, 1 week, 1 month, 6 months, and 12 months after the procedure. Postoperative examinations included uncorrected distance visual acuity (UDVA) and corrected distance visual acuity (CDVA) using a standard Snellen acuity chart at 6 m, manifest refraction, and slit lamp biomicroscopy.

Patients were observed for possible flap related complications including microfolds, epithelial ingrowth, interface haze, interface debris, infection, superficial punctate keratitis, and diffuse lamellar keratitis at each visit using a 6-grade classification system: trace, GD I-II (not visually significant), and GD III-V [11, 12].

### 2.6. Statistical Analysis

All treatments in both groups were performed as bilateral simultaneous LASIK. However, one eye was selected randomly (using computer generated random numbers) from each patient for statistical analysis. Outcome analysis was performed according to the Standard Graphs for Reporting Refractive Surgery [13]. Microsoft Excel 2010 (Microsoft Corporation, Redmond, WA) was used for data entry, and means and standard deviations were calculated for all parameters. Data were analysed using SPSS software (v 15; SPSS, Inc, Chicago, IL). Since the data was normally distributed, paired *t-*tests were used to calculate the statistical significance for comparison of postoperative parameters between the two study groups. A *p* value less than 0.05 was defined as statistically significant.

## 3. Results

Of the 328 eyes of 328 patients that underwent Femto-LASIK, 165 were treated with the MEL 90 and 163 with the Schwind Amaris 750S excimer laser. Of all patients, 56.8% were males and 43.2% were females. The mean follow-up duration of all patients from both groups was 12.2 ± 2.2 months (range 10.5 to 14.7 months). There were no statistically significant differences in preoperative manifest SE, cylinder, CDVA, keratometry, central corneal thickness, scotopic pupil size, intraoperative optical zone, mean flap thickness, ablation depth, and postoperative residual bed thickness) (RST) between the two groups (*p* values >0.05 or all parameters) (Table 1).

**Figure 1:**
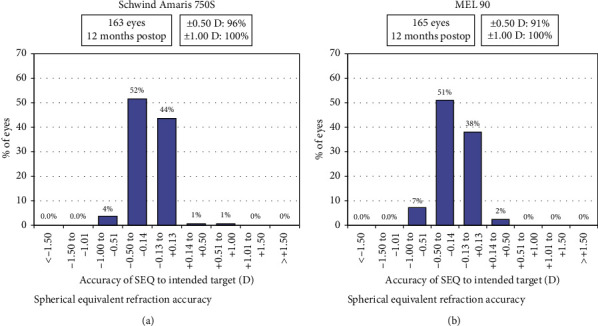
Spherical equivalent refraction accuracy of both groups at 12 months.

**Figure 2:**
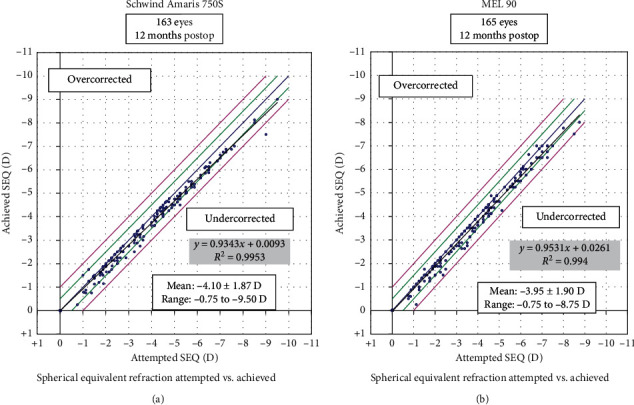
Attempted vs. achieved spherical equivalent refraction of both groups at 12 months.

The postoperative mean UDVA for the Amaris group was −0.10 ± 0.09 logMAR (range: −0.20 to 0.20), while for the MEL 90 group, it was −0.05 ± 0.07 logMAR (range: −0.20 to 0.10) (*p*=0.24). The accuracy of SE refraction within ±0.5 D was 96% eyes in the Amaris and 91% eyes in the MEL 90 group. However, all eyes in both the groups were within ±1.50 D of SE correction. The predictability curve gave a similar coefficient of determination values of 0.99 (Figures 1 and 2) and Table 2.

### 3.1. Safety

**Figure 3:**
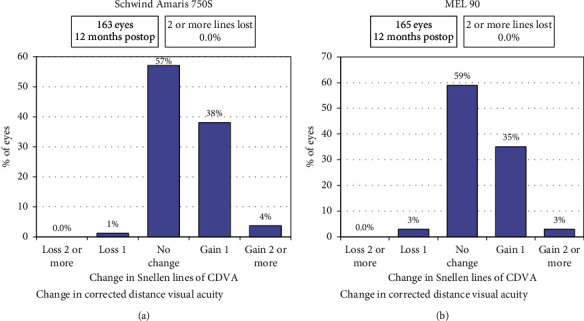
Safety (postop CDVA/preop CDVA) of both study groups at 12 months.

Safety index was defined as postoperative CDVA/preoperative CDVA. The mean safety indices of the Amaris and MEL 90 groups were 1.12 ± 0.16 (range 0.62 to 1.6) and 1.08 ± 0.15 (range 0.78 to 1.6), respectively (*p*=0.29).  Figure 3 shows the safety data of both the groups at 12 months. No eye lost more than 2 lines of CDVA in either of the groups (Figure 3).

### 3.2. Efficacy

**Figure 4:**
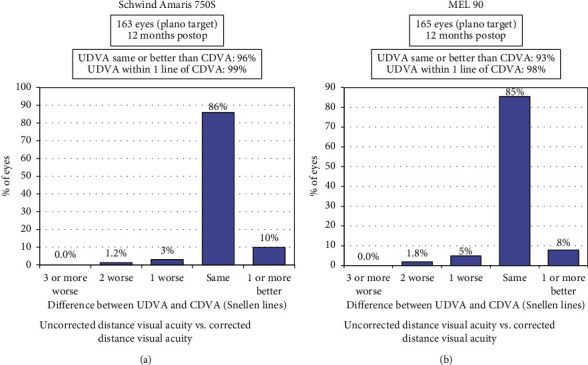
Uncorrected visual acuity vs. corrected visual acuity for both study groups at 12 months.

**Figure 5:**
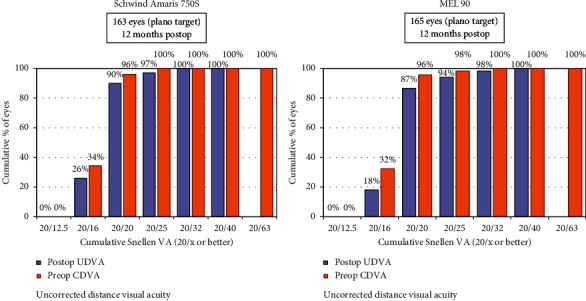
Cumulative uncorrected visual acuity of both study groups at 12 months.

Efficacy index was defined as postoperative UDVA/preoperative CDVA. The mean efficacy index of the Amaris group was 1.025 ± 0.10 (range 0.63 to 1.28), while that of the MEL 90 group was 1.00 ± 0.10 (range 0.5 to 1.25) (*p*=0.90). The percentage of eyes having postop UDVA same or better than preop CDVA was 96% in the Amaris group, versus 93% in the MEL 90 group (Figure 4). 18% of eyes in MEL 90 and 22% eyes in the Amaris group had cumulative UDVA of 20/16 or better (Figure 5).

### 3.3. Subgroup Analysis of Eyes with High Myopia (−6 D and above)

Both groups were comparable in terms of preop SE *p*=0.30. At the end of mean follow-up, too, there was no significant difference between the postop SE of the two study groups (*p*=0.66, −0.31 vs. −0.29 D for Schwind Amaris and MEL 90 groups, respectively). Similarly, postop UDVA, CDVA, Safety and Efficacy indices were comparable between the two groups (*p* > 0.05, for all parameters), Table 3.

### 3.4. Astigmatism Outcomes

**Figure 6:**
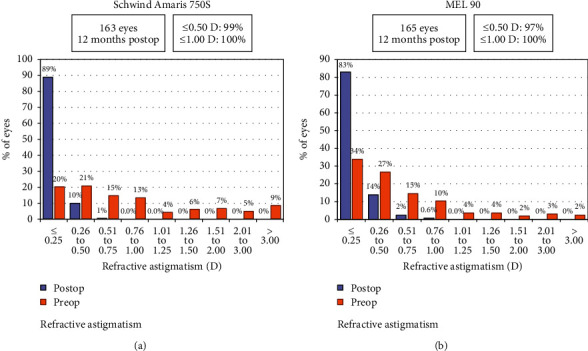
Refractive astigmatism distribution of both study groups at 12 months.

**Figure 7:**
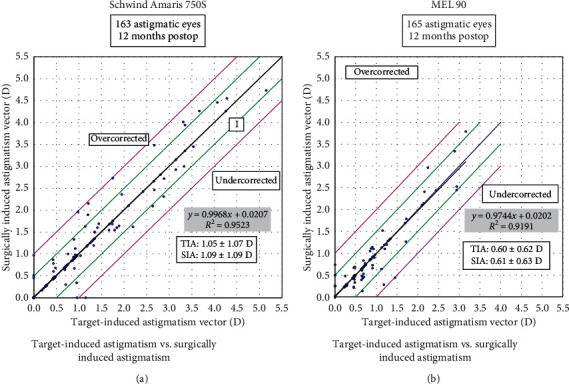
Target-induced astigmatism (TIA) vs. surgically induced astigmatism (SIA) for both study groups at 12 months.

**Figure 8:**
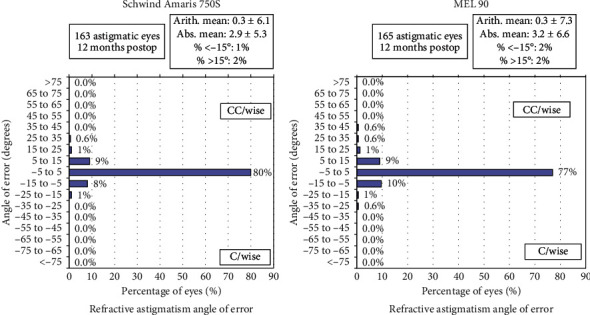
Refractive astigmatism angle or error distribution for both study groups at 12 months.

The mean postoperative cylinder was −0.13 ± 0.18 D (range: −0.75 to 0.5 D) in the Amaris group and −0.14 ± 0.21 D (range: −1.00 to 0.00 D) in the MEL 90 group (*p*=0.79). All eyes in both groups were within ±1.00 D of astigmatism (Figures 6 and 7). The angle of error (AE) graphs for both groups showed the majority of eyes (80% in the Amaris and 77% in MEL 90 group) having angle of error between −5 to +5 degrees (Figure 8).

### 3.5. Subgroup Analysis of High-Cylinder Eyes (>1 D)

We also performed a subgroup analysis of eyes with preop cylinder >1 D in both the groups, which showed preop astigmatism to be comparable (*p*=0.13). However, postop astigmatism was significantly lower in the Schwind Amaris group (−0.25 D) compared to the MEL 90 group (−0.39 D), (*p*=0.01). An undercorrection of 4% and 8% was observed in the Schwind Amaris and MEL 90 groups, respectively; however, the mean CI did not show any significant difference (Table 4).

### 3.6. Stability

**Figure 9:**
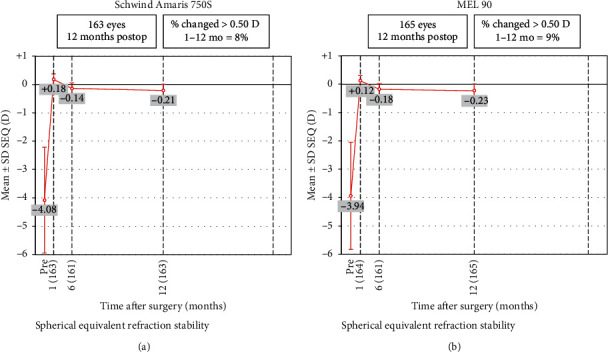
Stability of postoperative SE refraction at 1, 6, and 12 months for both study groups.

Both groups showed good stability of refraction at 1 year, compared to 1 month and 6 months, with slight residual myopia of −0.23 D and −0.21 D in MEL 90 and Amaris group, respectively (Figure 9).

### 3.7. Long-Term Complications

No vision threatening long-term complications such as diffuse lamellar keratitis, infectious keratitis, flap-folds, dislocations, epithelial ingrowth or, postoperative ectasia occurred within one year of the surgery in either of the groups. Four eyes of 2 patients in the MEL 90 group and both eyes of one patient in the Amaris group required enhancement at the last follow-up for significant residual refractive error due to progression of their myopia.

## 4. Discussion

The advantages of fast repetition rates and short ablation time, such as better patient safety and comfort, minimum risk of corneal dehydration, and reduced time of patient's eye fixation, have been reported in various studies [7, 8]. In the present study, we compared MEL 90 and Schwind Amaris 750S, which are currently the two fastest excimer lasers available for safety, efficacy, and predictability of outcomes obtained following Femto-LASIK at 12 months [7–10, 14–16].

It is pertinent to emphasize that the present study is a single surgeon study using the same standardized procedure, comparing the Schwind Amaris 750S, an established laser, with a newly installed MEL 90 laser. The results of our study showed that the MEL 90 wavefront-optimized excimer treatment was performed equally with the Amaris 750S platform in terms of postoperative UCVA, predictability, and safety and efficacy indices when aspherically optimized ablation profiles were used for MEL 90 except if cylinder over 1D was present.

The shorter treatment times with both lasers could be one of the main factors contributing to the comparable refractive predictability, as longer ablation time results in stromal bed drying, potentially affecting the treatment result. Although the Schwind Amaris^®^ operates at a higher frequency of 750 Hz, the intraoperative time taken to correct the same degree of myopia is slightly longer than MEL 90. It typically takes 1.3 seconds for treatment of 1.00 D myopia at an optical zone of 6.0 mm for the MEL 90, while the Amaris 750S takes 1.5 seconds for the same [7–10, 14–16]. This may be because of the differences in the spot sizes of both lasers, which are larger in MEL 90 (0.70 mm) compared to Schwind Amaris 750S (0.54 mm). Due to this, it requires firing less number of pulses per square area with MEL 90, thus, theoretically making the treatment slightly faster than Amaris 750s for correcting the same degree of refractive error. This, however, may not make much difference practically while correcting low to moderate degrees of myopia, as was the case in our study.

The Amaris 750S uses a dual-fluence concept, wherein approximately the first 80% of the ablation is performed with higher pulse energy, and the last 20% is completed with lower pulse energy to achieve the smoothest possible ablation surface using a small spot size of 0.54 mm and a super-Gaussian beam profile while reducing the thermal damage to the stromal bed [14, 15]. The MEL 90, on the other hand, utilizes uniform fluence throughout the ablation. However, it does not lead to increased heat production again due to its spot size being wider, requiring fewer pulses per square area, hence reducing the overall energy delivered to the cornea. Furthermore, its improved dynamic flow cone regulates the atmosphere more efficiently, preventing excess heat generation [7, 8].

Another aspect on which the accuracy of outcomes depends is the efficiency of eye-tracking during laser treatment. The more perfectly the eye is centred and the laser spots are positioned, the more precise the results of the refractive treatment are. For customized treatments and whenever astigmatism is greater than 1.00 D, compensation for possible cyclorotation has been suggested in various studies to achieve the intended outcome [17, 18]. Published reports have quoted an advantage in astigmatism control by the Amaris 750S system, which offers advanced eye-tracking technology with iris registration and static plus dynamic cyclotorsion compensation, including the rotating movement of the eye during the laser treatment [9, 10, 16, 19].

The MEL 90, on the other hand, has a 240 Hz video based infrared eye tracker, which also operates at 1,050 Hz, with active *x*- and *y*-axis and passive *z*-axis tracking [7, 8]. This, combined with iris registration from WASCA, also offers compensation of static cyclotorsion, occurring when the patient moves from upright to supine position. Dynamic cyclotorsion compensation, however, is not available in the current version of the laser.

The present study, in fact, showed no significant difference in postoperative astigmatism between eyes treated with the Amaris 750S versus those treated with the MEL 90, as the mean residual astigmatism was similar (−0.13 ± 0.18 D in Amaris 750S and −0.14 ± 0.21 D in MEL 90 group, respectively, *p*=0.79). This may suggest that good accuracy in astigmatism correction may still be achieved with a fast eye tracker and compensation of only static cyclotorsion, which forms for the major component of cyclotorsion [10]. However, subgroup analysis of high astigmatism eyes showed significantly lower postoperative astigmatism in the Schwind Amaris group, compared to the MEL 90 group, which may be attributed to the high-speed eye-tracking (pupil and limbus tracker with cyclotorsional tracking), as described earlier.

However, in a recently published study by Reinstein et al., they found a 12% overcorrection of astigmatism at 1 year for LASIK using the Triple-A ablation profile with the MEL 90 laser for mixed cylinder up to is −7.00 D, for which it was suggested that the results could be improved by the application of a nomogram [6]. Similarly, while evaluating outcomes of myopic LASIK with MEL 90 and triple-A profile, the same authors observed overcorrection of astigmatism at 3 months follow-up [7]. This is different from our results, wherein we observed an overall undercorrection of 7% (evident from a correction index of 0.93), which is expected at a follow-up period of 12 months. Also, we performed iris registration and compensation of cyclotorsional error for higher cylinders and used a frequency of 250 Hz for these eyes, which may also probably have influenced the results. However, from the clinical point of view, slight undercorrection is preferred above overcorrection.

It may be emphasized that 17/165 (10.3%) eyes requiring cyclotorsion compensation in MEL 90 group were treated with 250 Hz, whereas the rest were treated using 500 Hz frequency. This could have potentially influenced the cylinder accuracy and overall results, as stated above. However, the MEL 80 laser, using the repetition rate of 250 Hz has also been shown to provide excellent predictability in the previously published studies [20, 21], [22, 23], which may possibly explain the fairly comparable results with regard to astigmatism and overall accuracy between the two study groups. The mean UDVA in the Schwind Amaris treated eyes was better at one year postop. The difference, however, was not statistically significant.

To our knowledge, this is the first study comparing the outcomes of MEL 90® excimer laser with the Schwind Amaris® 750S for Femto-LASIK. The study demonstrated a tendency for slightly systematic better results with Amaris 750S, although in a nonsignificant manner. However, excellent safety and comparable results were observed in terms of postoperative UDVA, residual refraction, and efficacy with both lasers in a single surgeon setting, particularly applying to low astigmatism.

The retrospective and nonrandomized nature of this study may be a potential limitation. Therefore, a prospective, contralateral eye study with one eye of each patient assigned to each group would be more powerful for any further analysis of outcome measures. Nevertheless, the results reflect on the fact that newer and advanced technologies of excimer laser correction have certainly enhanced the overall safety and accuracy of outcomes with LASIK, resulting in better stability of outcomes and patient satisfaction.

## Figures and Tables

**Table 1:** Preoperative baseline characteristics of both study groups.

Parameter (mean ± SD) (range)	Schwind Amaris 750S	MEL 90	*p* value
Age(years)	33.00 ± 6.50 (23 to 53)	35.20 ± 10.50 (21 to 65)	0.33
Sph (D)	−3.53 ± 2.04 (−1.00 to −8.75)	−3.63 ± 1.80 (−1.00 to −8.80)	0.37
Cyl (D)	−1.13 ± 1.15 (0.00 to −6.00)	−0.74 ± 0.70 (0.00 to −3.50)	0.27
SE (D)	−4.10 ± 1.87 (−1.00 to −11.75)	−3.98 ± 1.896 (−1.00 to −10.50)	0.17
CDVA (logMAR)	−0.17 ± 0.02 (−0.2 to 0.00)	−0.053 ± 0.087 (−0.20 to 0.10)	0.07
CCT (*μ*m)	543 ± 28.6 (476 to 608)	536 + 32.4 (440 to 621)	0.31
Keratometry (D)	44.2 ± 2.3 (41.8–46.3)	43.7 ± 3.5(40.3–46.5)	0.43
Optical zone (mm)	6.50 ± 0.30 (6.10 to 7.00)	6.50 ± 0.20 (6.00 to 7.00)	1.00
Transition zone (mm)	1.20 ± 0.05 (1.00–1.50)	1.20 ± 0.03 (1.00–1.40)	0.80
RST(*μ*m)	369.70 ± 37.55 (302 to 484)	358.20 ± 46.54 (306 to 463)	0.22
Pupil size (mm)	6.14 ± 0.4 (5.6 to 6.7)	6.03 ± 0.3 (5.8 to 6.8)	0.30
Flap thickness (*μ*)	110 ± 11 (90 to 130)	110 ± 8.2 (90 to 120)	0.06
Flap diameter (mm)	7.90 ± 1.04 (7.50 to 8.10)	7.81 ± 1.05 (7.50 to 8.10)	0.09
Ablation depth (*μ*)	73.37 ± 27.48 (24 to 169)	68.00 ± 30.00 (20 to 131)	0.80

SE: spherical equivalent; CDVA: corrected distance visual acuity; CCT: central corneal thickness; RST: residual stromal thickness; SD: standard deviation.

**Table 2:** The postoperative visual and refractive results obtained at 1 year for both study groups.

Parameter (mean ± SD) (range)	Schwind Amaris 750S	MEL 90	*p* value
Sphere (D)	−0.15 ± 0.20 (−0.75 to 1.25)	−0.15 ± 0.21 (−1.00 to 0.50)	1.00
Cylinder (D)	−0.13 ± 0.18 (−0.75 to 0.5)	−0.14 ± 0.21 (−1.00 to 0.00)	0.79
SE (D)	−0.21 ± 0.22 (−0.87 to 1.25)	−0.23 ± 0.23 (−1.00 to 0.25)	0.29
UDVA (logMAR)	−0.10 ± 0.09 (−0.2 to 0.2)	−0.05 ± 0.07 (−0.20 to 0.10)	0.24
CDVA (logMAR)	−0.14 ± 0.06 (−0.2 to 0)	−0.09 ± 0.08 (−0.20 to 0.00)	0.24
Safety index	1.12 ± 0.16 (0.62 to 1.6)	1.08 ± 0.15 (0.78 to 1.6)	0.29
Efficacy index	1.02 ± 0.10 (0.63 to 1.28)	1.00 ± 0.10 (0.5 to 1.25)	0.90
AOE (arithmetic)	0.29 ± 6.05 (−24 to 30)	0.37 ± 7.34 (−30 to 40)	0.96
AOE (absolute)	2.87 ± 5.33 (0 to 30)	3.22 ± 6.60 (0 to 40)	0.82
CI	0.95 ± 0.33 (0 to 2.02)	0.93 ± 0.26 (0.21 to 1.48)	0.80

SD: standard deviation; SE: spherical equivalent; UDVA: uncorrected distance visual acuity; CDVA: corrected distance visual acuity; AOE: angle of error; CI: correction index.

**Table 3:** Subgroup analysis of eyes with high myopia (−6 D and above).

Parameter (mean ± SD) (range)	Schwind Amaris 750S (*n* = 26)	MEL 90 (*n* = 31)	*p* value
Preop SE	−6.19 ± 0.71 (−6.15 to −9.00)	−6.79 ± 0.70 (−6 to −8.75)	0.30
Postop SE	−0.31 ± 0.17 (0 to −0.625)	−0.29 ± 0.27 (−1.00 to 0.50)	0.66
Postop UDVA	−0.03 ± 0.09 (−0.2 to 0.20)	−0.03 ± 0.06 (0 to −0.20)	0.99
Postop CDVA	−0.11 ± 0.07 (0 to −0.20)	−0.08 ± 0.08 (0 to −0.20)	0.20
Safety index	1.18 ± 0.19 (1 to 1.60)	1.10 ± 0.18 (0.90 to 1.60)	0.11
Efficacy index	0.99 ± 0.09 (0.63 to 1.28)	0.99 ± 0.12 (0.625 to 1.25)	0.93

**Table 4:** Subgroup analysis of high-astigmatism eyes (1 D and above).

Parameter (mean ± SD) (range)	Schwind Amaris 750S (*n* = 38)	MEL 90 (*n* = 25)	*p* value
Preop cylinder	−2.34 ± 1.18 (−1.25 to −6.00)	−1.99 ± 0.75 (−1.25 to −3.50)	0.13
Postop cylinder	−0.25 ± 0.19 (0 to −0.75)	−0.39 ± 0.24 (0 to −1.00)	0.01^*∗*^
TIA	2.15 ± 1.33 (0.46 to 5.58)	1.73 ± 0.75 (0.43 to 3.16)	0.11
SIA	2.07 ± 1.11 (0.44 to 5.16)	1.60 ± 0.87 (0.28 to 3.79)	0.08
AOE-absolute	3.71 ± 4.27 (0 to 16)	3.20 ± 4.66 (0 to 15)	0.66
AOE-arithmetic	−0.5 ± 5.66 (−16 to 15)	0.875 ± 5.62 (−10 to 15)	0.35
CI	0.96 ± 0.23 (0 to 1.77)	0.92 ± 0.24 (0.23 to 1.32)	0.48

^∗^Independent t-test

## Data Availability

The data can be made available upon request from Dr. Sandhya R, who is in charge of the Ethics Committee of Nethradhama Eye Hospital (sandhyakrish@gmail.com).
